# Off-label use of tocilizumab to treat non-juvenile idiopathic arthritis in pediatric rheumatic patients: a literature review

**DOI:** 10.1186/s12969-018-0296-z

**Published:** 2018-12-14

**Authors:** Ju-Yang Jung, Moon-Young Kim, Chang-Hee Suh, Hyoun-Ah Kim

**Affiliations:** Department of Rheumatology, Ajou University of medical school, 164 Worldcup-ro, Yeongtong-gu, Suwon, 16499 Republic of Korea

**Keywords:** Tocilizumab, Interleukin-6, Autoimmune disease, Takayasu’s arteritis

## Abstract

Tocilizumab, an anti-interleukin-6 (IL-6) agent, is indicated as a treatment for several autoimmune or inflammatory diseases, including rheumatoid arthritis and juvenile idiopathic arthritis (JIA). IL-6 plays roles in both immune system dysregulation and inflammation, and thus efforts to extend the utility of tocilizumab in patients with autoinflammatory conditions are ongoing. Here, we survey the literature on the off-label use of tocilizumab in patients with juvenile-onset rheumatic diseases including juvenile systemic lupus erythematosus (SLE), juvenile dermatomyositis (DM), vasculitis, juvenile scleroderma, and other autoinflammatory diseases. There is no real evidence that tocilizumab is useful for patients with SLE and juvenile DM, but several cases of childhood Takayasu arteritis have experienced promising outcomes. In juvenile-onset scleroderma, for which no therapy that can halt disease progression is available, tocilizumab may stop progression and the associated functional impairment. Tocilizumab prevents systemic inflammation in patients with Kawasaki’s disease, but may develop coronary aneurysms. Tocilizumab has been used to treat several pediatric autoinflammatory diseases, including JIA-associated uveitis and Castleman’s disease. Further work in larger populations is necessary to confirm the effects of tocilizumab in patients with pediatric rheumatic diseases.

## Introduction

Tocilizumab, an anti-interleukin-6 (IL-6) agent, is indicated as a treatment for several autoimmune or inflammatory diseases, including rheumatoid arthritis (RA) and juvenile idiopathic arthritis (JIA). IL-6 plays roles in both immune system dysregulation and inflammation, and thus efforts to extend the utility of tocilizumab in patients with autoimmune conditions are ongoing. Here, we review IL-6 and tocilizumab, an IL-6 receptor blocker, and survey the literature on off-label tocilizumab use in pediatric patients with rheumatic diseases, such as juvenile-onset systemic lupus erythematosus (SLE), juvenile dermatomyositis (DM), vasculitis, and juvenile scleroderma.

### IL-6 and its receptor

IL-6 is produced by many types of cells including those of the immune system. It is barely detectable in healthy subjects, but its levels increase after infection or tissue damage [1]. IL-6 is released from macrophages, neutrophils, dendritic cells, and cluster of differentiation 4 (CD4) T cells, and it is associated with the clinical symptoms and signs of inflammation (such as fever and/or fatigue). IL-6 may induce bone marrow leukocytosis and thrombocytosis, stimulate hepatocytes to release C-reactive protein (CRP) and acute-phase markers affecting the erythrocyte sedimentation rate (ESR), activate neutrophils and macrophages, and cause T-helper cells to differentiate into type 17 T-helper (Th17) cells [2]. On the other hand, it inhibits T-regulatory (Treg) cells, and the resulting Th17/Treg imbalance compromises immunological tolerance [3]. It also promotes the differentiation of B cells into antibody-producing cells and CD8-positive T cells into cytotoxic T cells [1, 4, 5]. It acts via a ubiquitous receptor (IL-6R) composed of two chains, IL-6R and gp130; the latter transduces the IL-6 signal into cells [6–8]. IL-6R expression is restricted to certain cells including leukocytes and hepatocytes, but gp130 is expressed in almost all cells [9]. A soluble form of IL-6R is found in human serum, and an IL-6/soluble IL-6R complex can bind to cells lacking IL-6R, transducing the IL-6 signal via gp130 without any need for transmembrane IL-6R [10]. The IL-6 signal activates “signal transducer and activator of transcription 3,” triggering the secretion of many proteins including CRP and haptoglobin, which mount inflammatory reactions. IL-6 plays a critical role in terms of coordination of the innate and adaptive immune responses and the switch from acute to chronic inflammation, and is considered a useful target in a variety of autoimmune diseases.

### Tocilizumab

Tocilizumab, a humanized anti-IL-6R monoclonal antibody, is the first drug to be found to successfully block the IL-6 signal; the drug binds to both cell-surface and soluble IL-6R [11]. Tocilizumab was designed by a group led by Tadamitsu Kishimoto, and was approved by the U.S. Food and Drug Administration (FDA) in 2010 for the treatment of RA. It alleviates inflammation and prevents bone damage associated with RA, moderating disease activity (including joint tenderness and swelling) and disease activity scores within 1 month after commencement [12]. Furthermore, the FDA approved the drug as a treatment for systemic JIA in 2011, for polyarticular JIA in 2013, and for giant cell arteritis in 2017. Tocilizumab effectively treats refractory adult-onset Still’s disease, the adult form of systemic JIA, polymyalgia rheumatica, and various manifestations of giant cell arteritis via its steroid-sparing effects [13–15]. Efforts to explore and extend the utility of tocilizumab are ongoing; off-label tests in patients with other systemic inflammatory diseases are underway.

## Methods

We screened PubMed and MEDLINE using the terms “tocilizumab” AND “pediatric” OR “autoinflammatory”, and “juvenile”. Among the 466 retrieved articles published between November 1994 and August 2018, 256 articles reporting on the use of tocilizumab in patients with JIA were excluded. Randomized and nonrandomized controlled trials, case-control studies, cohort studies, and case series and single case reports involving pediatric patients treated with tocilizumab were included. The remaining 210 articles were reviewed, of which 71 articles described pediatric diseases treated with tocilizumab in patients not diagnosed with JIA.

Information related to tocilizumab treatment, including patient age at the time of diagnosis, sex, age at the time of tocilizumab therapy, disease duration before tocilizumab therapy, tocilizumab dose and administration route, previous use of immunosuppressive agents, doses of glucocorticoids before and after tocilizumab treatment, concurrent treatments, response to tocilizumab therapy, and adverse events related to tocilizumab infusion, was collected.

## Results

### SLE

SLE is a systemic autoimmune disease involving the skin, joints, kidneys, and other organs via autoantibody production and the formation of immune complexes. Childhood-onset SLE is rare (0.3–0.9/100,000 subjects); the median age at onset is about 11 years [16]. Pediatric SLE exhibits a more severe course than adult SLE and is associated with a higher rate of morbidity [17]. Serum IL-6 levels are elevated and correlated with disease activity in patients with lupus [18]. IL-6 administration to lupus-prone mice increases autoantibody production and accelerates the progression of glomerulonephritis [19]. In an open-label phase I study, tocilizumab improved the disease scores of patients with moderately active SLE, but neutropenia and infection developed in a dose-dependent manner [20]. Nonetheless, several case reports have described the successful use of tocilizumab in the treatment of SLE flares in adult patients [21–23]. Combined cyclophosphamide and tocilizumab treatment improved massive pericarditis and glomerulonephritis in a patient with refractory SLE [24]. However, the use of tocilizumab to treat pediatric patients with SLE has not been well examined. In a previous case report, we described the results of tocilizumab therapy in a 20-year-old patient with SLE (diagnosed at the age of 13 years). Prior to therapy, she experienced recurrent flare-ups, with fever, lymphadenopathy, and erosive inflammatory arthritis; she was negative for rheumatoid factor and anti-citrullinated protein antibody. After tocilizumab was commenced, her arthritis and inflammatory markers resolved and her glucocorticoid dose was successfully reduced [25].

### Juvenile DM

Juvenile DM is the most common form of myositis in children, characterized by symmetrical, proximal muscle weakness with typical rashes (Gottron’s papules and the heliotrope rash) [26, 27]. The rashes reflect an immune system-mediated vasculopathy involving the periungal capillaries. The median age at onset is 7.5 years, and 24–40% of patients recover within 2 years. Serum levels of IL-6 increase in patients with either adult or juvenile DM, and are associated with disease activity [28–30]. A case report described an adult patient with concomitant DM, SSc, and RA whose disease activity improved with tocilizumab therapy [31]. In one case, a 12-year-old patient with juvenile DM, treated with glucocorticoids and methotrexate (MTX), relapsed with Raynaud’s phenomenon, sclerodactyly, and polyarthritis [32]. After several drugs failed, tocilizumab was initiated, but improved joint manifestations only.

### Vasculitis

Takayasu arteritis (TA) is a granulomatous vasculitis involving the aorta and its main branches, and occurs in both adult and children [33]. TA manifests as constitutional symptoms such as weight loss and anorexia, and progresses to claudication and hypertension caused by pathological vascular narrowing or occlusion. Glucocorticoids are the mainstay of therapy; immunosuppressive agents including MTX and cyclophosphamide play roles as glucocorticoid-sparers [34]. In TA patients, IL-6 is synthesized by activated dendritic cells in the walls of blood vessels, activating CD4+ T cells and inducing the maturation of Th1 and Th17 cells [35]. IL-6 levels are elevated in serum and aortic tissue. Several studies have found that tocilizumab is effective for adults with TA [36, 37]. Table 1 lists the results of tocilizumab treatment in patients with childhood TA. In one study, a 3-year-old girl with fever, a cardiac murmur, and absence of the right radial pulse was diagnosed with TA because of a high level of CRP, thickening of the aortic-arch wall, and complete occlusion of the common carotid arteries [38]. High-dose prednisone and MTX suppressed the disease, but the condition was repeatedly reactivated when the glucocorticoid was tapered. Etanercept and infliximab were ineffective; then she received several immunosuppressive agents and achieved partial disease control that was glucocorticoid-dependent. Tocilizumab was commenced when she developed a new relapse associated with elevated inflammatory marker levels and retrosternal pain. Disease activity was reduced and the prednisone dose was lowered after 15 months. Four pediatric TA cases treated with tocilizumab have been reported; the clinical and laboratory responses were good [39]. In that study, all patients with active disease achieved an inactive state (no new vascular lesion) after 3 months of tocilizumab injections. In another case, a 13-year-old girl with fever, weight loss, mesenteric claudication, and left shoulder pain was diagnosed with TA based on fluorodeoxyglucose uptake (evident via positron emission tomography-computed tomography [PET-CT]) by the aortic arch of the descending aorta, the left internal carotid artery, and the left subclavian artery [40]. High-dose corticosteroids with MTX were initially effective, but the disease relapsed. Infliximab therapy failed and thus tocilizumab was commenced at 8 mg/kg monthly. The symptoms and inflammatory marker levels were improved and PET-CT revealed no FDG uptake after 3 months of therapy. Several other cases in which pediatric patients with TA were treated successfully with tocilizumab have been reported [41, 42]. Two pediatric patients with in-stent restenosis had stable disease activity during tocilizumab therapy, although disease reactivation occurred in one patient 3 months after tocilizumab withdrawal [42].

**Table 1:** Off-label use of tocilizumab (TCZ) in pediatric patients with Takayasu arteritis

Authors, year (ref.)	Sex	Age at onset, years	Age at TCZ, years	Manifestations	Previous therapy	Dose of TCZ	Duration of TCZ, months	Adverse effect of TCZ	
Bravo Mancheno et al., 2012 [38]	F	3	5	Wall thickening, occlusion	GC, MTX, ETN, IFX, CYC, MMF	8 mg/kg /2–3 weeks	> 24	No	
Batu et al., 2017 [39]	F	4	9	Stenosis	GC, CYC, MTX, AZA	8 mg/kg /4 weeks	7–13	No	
	F	13	15	Aneurysms					
	F	16	16	Graft, stenosis and aneurysms	GC, CYC, MTX				
					GC, CYC, ETN				
	F	16	16	Wall thickening	GC				
Decker et al., 2018 [40]	F	13	15	Panaortitis	GC, MTX, IFX	8 mg/kg /4 weeks	18	No	
Canas et al., 2014 [41]	F	12		Wall thickening	GC	8 mg/kg /4 weeks	18	No	
	F	14		Wall thickening	GC, MTX		12		
Goel et al., 2013 [42]	F	13	13	In-stent restenosis	GC, MMF	8 mg/kg /4 weeks	6	No serious AE	
				In-stent restenosis	GC, MMF				
	F	14	15						

*GC* glucocorticoids, *MTX* methotrexate, *ETN* etanercept*, IFX* infliximab, *CYC* cyclophosphamide, *MMF* mycophenolate mofetil, *AZA* azathioprine

### Scleroderma

Systemic sclerosis (SS, also termed scleroderma) is a systemic autoimmune disease characterized by autoantibody production, inflammation of skin and soft tissue, and fibrosis. Juvenile scleroderma includes juvenile localized scleroderma and juvenile systemic scleroderma [43]. Most current drugs including MTX are ineffective against both juvenile and adult scleroderma. The peripheral B cells of adults with SS secrete IL-6, the serum levels of which correlate with disease activity and the extent of internal organ involvement [44, 45]. In a phase 2, randomized controlled trial on adults with SS, tocilizumab failed to improve skin thickening significantly but slowed the decline in forced vital capacity [46]. Two children with pansclerotic morphea (a severe subtype of localized scleroderma) refractory to various immunosuppressive agents were treated with tocilizumab [47]; disease activity was controlled and progression ceased. Five patients aged 6–13 years with localized scleroderma refractory to immunosuppressants (including MTX and mycophenolate mofetil) or etanercept were started on tocilizumab [48]. Disease activity, tissue damage, and quality-of-life improved after 3 months; all patients tolerated tocilizumab for 12–25 months without any serious adverse reactions. No drug of choice is yet available for juvenile scleroderma; tocilizumab may control inflammation and stop progression.

### Autoimmune encephalitis

Autoimmune encephalitis (AE) is an inflammatory disorder of the central nervous system [49, 50]. It commonly presents with the sudden onset of memory deficit, altered mental status, psychiatric symptoms and seizure, cerebrospinal fluid pleocytosis, and magnetic resonance imaging features of encephalitis. Although the discovery of pathogenic autoantibodies has changed the diagnosis and treatment of AE, diagnostic and therapeutic challenges remain. Immunosuppressive therapy, including high-dose glucocorticoids and cyclophosphamide, is used to minimize permanent neurological damage. Thirty adult patients with inadequate clinical responses to rituximab therapy received tocilizumab, which resulted in favorable clinical responses in all cases [51]. A case series described two pediatric patients with seizure and one with ataxia and cognitive dysfunction with disease refractory to glucocorticoids, intravenous immunoglobulin, and rituximab [52]. Following deterioration of their neurological symptoms, they were treated with tocilizumab, which resulted in symptom resolution after 2 to 4 weeks. The successful use of tocilizumab supports its administration to pediatric patients with refractory and aggressive autoimmune encephalitis.

### Kawasaki’s disease

Kawasaki’s disease is an acute, self-limiting vasculitis affecting the medium-sized blood vessels of infants and children [53, 54]. Although high-dose, intravenous immunoglobulin (IVIG) treatment is effective, 25% of children develop coronary artery aneurysms. A cytokine storm including tumor necrosis factor (TNF)-α, IL-6, and granulocyte colony-stimulating factor is evident in patients in whom coronary artery aneurysms are developing [55]. Four patients, two 1-year-old girls and 8- and 4-year-old boys with IVIG-resistant Kawasaki’s disease, were treated with tocilizumab [56]. All recovered from fever and leukocytosis, and inflammatory marker levels fell, but the two girls developed progressive giant coronary artery aneurysms. IL-6 plays a role in tissue regeneration; tocilizumab may disrupt this process by blocking the action of vascular endothelial growth factor. These cases suggest that tocilizumab may trigger new-onset coronary aneurysms.

### JIA-associated refractory uveitis

Tocilizumab effectively treats not only systemic or polyarticular JIA, but also JIA-associated uveitis [57–60], which presents as chronic nongranulomatous anterior uveal inflammation causing permanent ocular damage and visual loss. Several case reports and multicenter studies have reported that tocilizumab is valuable in patients with JIA-associated refractory uveitis (Table 2) [58, 59, 61, 62]. One study used tocilizumab to treat JIA-associated uveitis refractory to immunosuppressive drugs and anti-TNF agents [58]. Overall, 79.2% of patients exhibited improvements in anterior chamber cell numbers; steroid-sparing was possible. However, serious complications including severe autoimmune thrombocytopenia and pneumonia have been reported. A recent study described four patients with long-term, JIA-associated chronic anterior uveitis who exhibited good sustained responses on intravenous tocilizumab [63]. However, they experienced early flares after switching to subcutaneous tocilizumab. Ocular disease was more readily controlled by intravenous rather than subcutaneous tocilizumab.

**Table 2:** Off-label use of tocilizumab in pediatric patients with JIA-associated uveitis

Authors, year (ref.)	Sex	Age at onset, years	Age at TCZ, years	Previous therapy	Dose of TCZ	Efficacy of TCZ	Duration of TCZ	Adverse effect of TCZ
Tsang et al., 2014 [60]	M	3	12	GC, MTX, ADA, RTX	8 mg/kg/2–4 weeks	Quiescence of uveitis	20 weeks	Neutropenia when infused every 2 weeks
Tappeiner et al., 2016 [58]	F:M 14:3		15.3 ± 6.9	GC, MTX, ≥1 TNF-α inhibitor		Inactive in 10, persisted in 7	5.7 months	No
Calvo-Rio et al., 2017 [57]	F:M 21:4		18.5 ± 8.3 (8–38)	GC, MTX, IFX, ETN, ADA, GLM, ABA, ANK, RTX	8 mg/kg/2, 4, 8 weeks	Inactive in 19 cases	5.5–24 months	Thrombocytopenia (1), pneumonia, thrombocytopenia, and hemolytic anemia (1), Viral conjunctivitis and bullous impetigo (1)
					162 mg/week SC			
Quesada-Masachs et al., 2017 [62]	F	1.9	14	MTX, leflunomide, ADA	8 mg/kg/month switching to 162 mg/week SC	Ocular flare after 6 months	6 months	No

*TCZ* tocilizumab, *GC* glucocorticoids, *MTX* methotrexate, *ADA* adalimumab, *RTX* rituximab, *TNF* tumor necrosis factor, *IFX* infliximab, *ETN* etanercept, *GLM* golimumab, *ABA* abatacept*, ANK* anakinra

### Castleman’s disease

Increased IL-6 levels have been observed in Castleman’s disease, which is a poorly understood lymphoproliferative disorder driven by proinflammatory hypercytokinaemia [64]. One study found that tocilizumab was efficacious in two children with multicentric Castleman’s disease who developed sustained fever after treatment with an IL-1 agonist or IVIG [65]. A 21-month-old boy with multicentric Castleman’s disease responded incompletely to initial tocilizumab treatment, but improved after subsequent chemotherapy [66]. In another case, a 16-year-old male was diagnosed with multicentric Castleman’s disease associated with abdominal pain, fatigue, weakness, fever, and night sweats [67]. He exhibited sustained remission after multi-agent chemotherapy and maintenance tocilizumab therapy, which suggests that tocilizumab is useful for treating multicentric Castleman’s disease.

### Amyloid a amyloidosis

Secondary amyloidosis may develop in patients with chronic inflammatory diseases, including JIA, invading the kidneys, liver, heart, gastrointestinal system, and autonomic nervous system [68]. Tocilizumab therapy was reported to improve renal amyloidosis-related systemic-onset JIA in three female patients aged 26, 29, and 19 years [69, 70]. These three patients had been diagnosed with systemic JIA, at 14, 6, and 9 years of age, respectively, and they were treated with MTX, cyclophosphamide, or etanercept with glucocorticoids before the diagnosis of amyloidosis. One of the patients, with gastrointestinal and renal involvement, had improved levels of inflammatory markers and symptom reduction after tocilizumab infusion. In another patient, amyloidosis developed during a treatment regimen of 8 mg tocilizumab/kg every 4 weeks; the tocilizumab dose was thus increased to 8 mg/kg every 2 weeks. Clinical improvement occurred within 3 months.

### Cytokine release syndrome

Cytokine release syndrome (CRS) is a serious response of patients with hematological malignancies to novel T cell-engaging therapies [71, 72]. High-level immune activation, including T cell activation and cytokine level elevation, results in fever and other manifestations, including cardiac dysfunction, toxicity, renal or hepatic failure, and disseminated intravascular coagulation. Tocilizumab was used to control severe symptoms in two pediatric patients with relapsed acute lymphoblastic leukemia (ALL) who developed CRS after chimeric antigen receptor-modified T cell therapy [73]. The 7-year-old male and 11-year-old female were each treated with a single dose of 8 mg tocilizumab/kg, and both patients had clinical responses that included improved hemodynamic status, blood cell counts, and inflammatory markers. Tocilizumab is recommended for patients with grade 3 and 4 CRS, with life-threatening symptoms requiring aggressive intervention. In a phase I/IIa clinical trial of chimeric antigen receptor-modified T cell therapy for relapsed/refractory ALL, 13 of 39 patients with CRS had cardiovascular dysfunction and were thus treated with tocilizumab. Four patients received more than one dose of tocilizumab, but all patients recovered from the CRS [74].

### Other autoinflammatory conditions

Systemic autoinflammatory disorders are closely associated with gene mutations and are caused by the dysregulation of innate immunity. TNF receptor-associated periodic syndrome (TRAPS) is an autosomal dominant inherited autoinflammatory disorder caused by mutations in the gene encoding the TNF receptor. It is characterized by recurrent and prolonged fever, myalgia, arthralgia, erysipelas, and serositis. A 4-year-old boy diagnosed with TRAPS related to a heterozygous variant in the *TNFRSF1A* gene had already received glucocorticoids, anti-IL-1β monoclonal antibody, TNF inhibitor (infliximab), and tocilizumab based on a previous diagnosis of systemic JIA [75]. After the DNA mutation was identified, he received a tocilizumab infusion, which resulted in the sustained remission of all symptoms for at least 2 years.

H syndrome, also called pigmentary hypertrichosis and non-autoimmune insulin-dependent diabetes mellitus (PHID) syndrome, is an autosomal recessive genetic disorder whose features include the childhood onset of skin lesions associated with autoantibody-negative insulin-dependent diabetes mellitus [76]. The condition, caused by a mutation in SLC29A3, is characterized by severe autoinflammation and the increased production of pro-inflammatory cytokines. Four patients with PHID whose disease manifestations had emerged at the age of 2–5 years were treated with tocilizumab [77, 78]. In the three patients who received a TNF inhibitor after MTX or cyclosporin, the symptoms continued. Tocilizumab therapy, as an intravenous infusion administered at a dose of 8 mg/kg or injected subcutaneously at a dose of 10.8–12 mg/kg every 2 weeks, was started when the patients reached the ages of 3–19 years, to treat the severe ongoing systemic inflammation.

A 15-year-old girl presented with exertional dyspnea, hemoptysis, episodic fever, oropharyngeal ulcer, arthritis, and vasculitic rash. Based on the clinical work-up, including lung biopsy, she was diagnosed with an undifferentiated autoinflammatory disease with interstitial pneumonitis [79]. Infusions of IL-1 receptor antagonist improved the rash, oral ulcer, and arthritis, but aggravated the patient’s respiratory symptoms. After tocilizumab therapy, amelioration of her extrapulmonary symptoms, oxygen saturation, and flares of dyspnea was achieved, accompanied by a decrease in parenchymal ground-glass changes, as determined by computed tomography.

### Grave’s disease

Graves’ ophthalmopathy occurs mainly in Graves’ hyperthyroidism, which is caused by thyroid-stimulating hormone receptor-stimulating antibodies produced by lymphocytes in the thyroid gland or lymph nodes [80]. It initially involves the extraocular muscles, but then expands to the orbital fat and subcutaneous tissue, leading to swelling and redness of the eyelids and conjunctiva, exophthalmos, and double vision. Disease refractory to systemic glucocorticoids and surgical orbital decompression results in corneal ulceration and the loss of visual acuity. Immunosuppressants have been prescribed in severe cases, given the inflammatory pathogenesis of thyroid eye disease. Orbital fibroblasts produce excessive IL-6, which recruits and activates T cells to induce the synthesis of hydrophilic glycosaminoglycans, with effects on other immune cells. The improvement of several adult cases of severe thyroid eye disease following tocilizumab therapy has been reported [81, 82]. Moreover, a randomized clinical trial demonstrated the meaningful efficacy of tocilizumab in 32 patients with moderate to severe corticosteroid-resistant Graves’ orbitopathy [83]. Although the use of tocilizumab in pediatric patients with Grave’s ophthalmopathy has not been reported, these results suggest that pediatric Graves’s ophthalmopathy will be responsive to tocilizumab.

## Conclusion

IL-6 is a key driver of systemic autoimmune diseases; tocilizumab-mediated IL-6 blockade is effective for children with systemic or polyarticular JIA. Table 3 summarizes the effects of tocilizumab on pediatric rheumatic diseases other than JIA. Little evidence of any therapeutic effects of tocilizumab in patients with SLE and juvenile DM is available, but several cases of childhood TA have exhibited promising responses to treatment. No effective therapy for juvenile scleroderma is available; tocilizumab may stop progression and functional impairment. Tocilizumab is active against systemic inflammation in patients with Kawasaki’s disease but coronary aneurysms may nonetheless develop. This drug might prevent the loss of visual acuity in patients with JIA-associated uveitis and allow sustained clinical remission in patients with multicentric Castleman’s disease. The use of tocilizumab should be based on consideration of the actual benefits of the drug, in view of possible adverse events, including serious infection.

**Table 3:** Off-label use of tocilizumab in pediatric patients with non-JIA rheumatic diseases except Takayasu arteritis and JIA-associated uveitis

Disease	*n*	Age, years	Efficacy of TCZ	Dose of TCZ	Adverse effect of TCZ	References
Juvenile SLE	1	13	Improved arthritis and inflammatory markers Reduction of GC dose	400 mg/month	No	[21]
Juvenile dermatomyositis	1	12	Improved arthritis	8 mg/kg/4 weeks	No	[28]
Takayasu arteritis	12	3–16	Improved symptoms and inflammatory marker Reduction of GC dose No FDG uptake	8 mg/kg/3–4 weeks	No	[34–38]
Juvenile localized scleroderma	7	6–13	Improved disease activity and inhibition of progression	≥30 kg: 8 mg/kg < 30 kg: 12 mg/kg every 4 weeks	No	[47, 48]
Autoimmune encephalitis	3	10, 12, 15	Improved neurological symptoms without recurrence	8–12 mg/kg/4 weeks	No	[51]
Kawasaki’s disease	4	1–8	Improved symptoms and inflammatory markers	8 mg/kg once	Giant coronary artery aneurysm (2)	[55]
JIA-associated uveitis		8–38	Improved visual acuity Reduction of GC dose	8 mg/kg/4 weeks	Thrombocytopenia (1), pneumonia, thrombocytopenia, and hemolytic anemia (1), viral conjunctivitis and bullous impetigo (1)	[57, 58, 60, 62]
Multicentric Castleman’s disease	4	1–16	Sustained remission	8 mg/kg/2 weeks 10 mg/kg three times 8 mg/kg/2 weeks	Herpes zoster (1)	[64–66]
Amyloid A amyloidosis	3	19, 26, 29	Improved symptoms and inflammatory markers	8 mg/kg/2–4 weeks	No	[68, 69]
Cytokine release syndrome (CRS)	15	7–16	Improved symptoms and inflammatory markers	8 mg/kg 1–3 times	No	[72, 73]
Tumor necrosis factor receptor-associated periodic syndrome (TRAPS)	1	4	Improved symptoms and inflammatory markers	8 mg/kg/month	No	[74]
H syndrome (PHID)	4	3–19	Improved symptoms and inflammatory markers	IV: 8 mg/kg SQ: 10.8–12 mg/kg every 2 weeks	No	[76, 77]
Undifferentiated autoinflammatory disease with interstitial pneumonitis	1	15	Improved symptoms and reduction of ground-glass opacity on CT	8 mg/kg/month	No	[78]

*JIA* juvenile idiopathic arthritis, *SLE* systemic lupus erythematosus, *TCZ* tocilizumab, *GC* glucocorticoids, *IV* intravenous, *SQ* subcutaneous, *CT* computed tomography

This literature review supports the use of tocilizumab therapy as one option in the treatment of several pediatric rheumatic diseases, not limited to JIA. Although the role of tocilizumab in the treatment of systemic autoimmune diseases may be insufficient, IL-6 is a protagonist of the inflammasome-mediated autoinflammatory response. Considering the mechanism of IL-6 and the effects of tocilizumab, the drug can be used in several disorders in which autoinflammatory reactions are the main pathogenesis. However, further studies, including well-designed clinical trials, are needed to confirm the effects of tocilizumab and to evaluate its safety profile in larger populations of pediatric patients with rheumatic diseases.

## Abbreviations

ALL: Acute lymphoblastic leukemia
CD4: Cluster of differentiation 4
CRP: C-reactive protein
CRS: Cytokine release syndrome
DM: Dermatomyositis
ESR: Erythrocyte sedimentation rate
FDA: Food and Drug Administration
IL-6: Interleukin-6
IVIG: Intravenous immunoglobulin
JIA: Juvenile idiopathic arthritis
MTX: Methotrexate
PET-CT: Positron emission tomography-computed tomography
PHID: Pigmentary hypertrichosis and non-autoimmune insulin-dependent diabetes mellitus
RA: Rheumatoid arthritis
SLE: Systemic lupus erythematosus
SS: Systemic sclerosis
TA: Takayasu arteritis
Th17: Type 17 T helper
TNF-α: Tumor necrosis factor-α
TRAPS: TNF receptor-associated pediatric syndrome
Treg: T-regulatory
